# What Players of Virtual Reality Exercise Games Want: Thematic Analysis of Web-Based Reviews

**DOI:** 10.2196/13833

**Published:** 2019-09-16

**Authors:** Nuša Faric, Henry W W Potts, Adrian Hon, Lee Smith, Katie Newby, Andrew Steptoe, Abi Fisher

**Affiliations:** 1 Behavioural Science and Health University College London London United Kingdom; 2 University College London London United Kingdom; 3 Six to Start LSD Accountants London United Kingdom; 4 The Cambridge Centre for Sport and Exercise Sciences Anglia Ruskin University Cambridge United Kingdom; 5 Coventry Univesity Coventry United Kingdom

**Keywords:** virtual reality, exercise, video games, sedentary lifestyle, weight loss, behavior, obesity, sports

## Abstract

**Background:**

Physical activity (PA) is associated with a variety of physical and psychosocial health benefits, but levels of moderate-to-vigorous intensity PA remain low worldwide. Virtual reality (VR) gaming systems involving movement (VR exergames) could be used to engage people in more PA.

**Objective:**

This study aimed to synthesize public reviews of popular VR exergames to identify common features that players liked or disliked to inform future VR exergame design.

**Methods:**

We conducted a thematic analysis of 498 reviews of the 29 most popular exergames sold in the top 3 VR marketplaces: Steam (Valve Corporation), Viveport (Valve Corporation), and Oculus (Oculus VR). We categorized reviews as positive and negative as they appeared in the marketplaces and identified the most common themes using an inductive thematic analysis.

**Results:**

The reviews were often mixed, reporting a wide variety of expectations, preferences, and gaming experiences. Players preferred highly realistic games (eg, closely simulated real-world sport), games that were intuitive (in terms of body movement and controls), and games that provided gradual increases in skill acquisition. Players reported feeling that they reached a high level of exertion when playing and that the immersion distracted them from the intensity of the exercise. Some preferred features included music and social aspects of the games, with multiplayer options to include friends or receive help from experienced players. There were 3 main themes in negative reviews. The first concerned bugs that rendered games frustrating. Second, the quality of graphics had a particularly strong impact on perceived enjoyment. Finally, reviewers disliked when games had overly complex controls and display functions that evoked motion sickness.

**Conclusions:**

Exergames prove to be a stimulating avenue for players to engage in PA and distract themselves from the negative perceptions of performing exercise. The common negative aspects of VR exergames should be addressed for increased uptake and continued engagement.

## Introduction

### Background

There is a large body of evidence showing that regular and sustained participation in physical activity (PA) aids in the prevention and management of noncommunicable disease [1-5]. Age-specific PA recommendations have been formulated, for example, the United Kingdom (UK) government recommends at least 150 min per week of moderate-to-vigorous PA for adults and 60 min per day for children and adolescents aged 5 to 18 years [6]. However, global population levels of PA are insufficient [6-8]. The UK has particularly low levels of PA compared with other European countries [9]. Objective measures of PA in 4507 adults aged above 16 years showed that only 6% of males and 4% of females met the recommended guidelines for PA [10]. Furthermore, 50% of males and 58% of females were classified as *inactive* [10]. Levels of inactivity are particularly high among socioeconomically deprived groups [11,12]. The US Department of Health and Human Services reported that less than 5% of American adults engage in 30 min of PA every day and only one-third reach the recommended PA levels in a week [13]. An increase in sedentary lifestyle and insufficient PA has also been observed in other countries such as Australia [14], India [15], and China [16]. Interventions to increase levels of PA in the population are urgently required.

Gaming is a recreational activity regularly performed by 2 to 3 billion people worldwide across socioeconomically deprived groups and by males and females [17,18]. The popularity and nature of gaming makes it a potential avenue for a population health intervention [19]. A market research report by Limelight Networks in 2018 found that gamers around the world spent an average 6 hours in playing each week [20]. Games requiring bodily movement (exergames) offer a potential to increase PA and reduce sitting time or replace sedentary screen time [21]. Early generation exergames such as Dance Dance Revolution released in 1998 and Nintendo’s Wii Fit released in 2007, (which sold over 22 million copies worldwide) and, more recently, Pokémon Go (downloaded over 800 million times) are examples of exergames that have had huge commercial success [22,23]. Empirical studies of previous generation exergames have shown that they can achieve PA of at least moderate intensity and can increase energy expenditure up to 300% above resting levels [23]. In addition, a meta-analysis of 35 studies in young people found that they achieved a similar level of activity in field-based sports and enhanced reported enjoyment of, and intrinsic motivation for, PA [24]. Exergaming has also been positively associated with psychological well-being [25,26]. Randomized controlled trials in children [27], preadolescents [28], adolescents [29,30], and adults [31] found that exergames supported weight loss and increased fitness. However, trials have not been able to confirm exactly which psychological or social factors might lead to long-term engagement [21,25,26].

Virtual reality (VR) has the potential to enhance the impact of an exergaming intervention. The most prominent feature of VR is high-quality virtual realism or immersion, coupled with body tracking, where the person feels fully present or located in the virtual world [32,33]. In exergames, presence is not always required, but in VR, full presence is required and, as such, psychological processes may potentially differ and even influence the performance or affective states of players [33]. There is evidence from laboratory studies that immersive VR exergaming is more engaging than standard exercise and may distract participants from exertion. For example, one study involving 88 university staff and students found that although the heart rate was higher in VR than a standard exercise condition, participants reported feeling less tired and had higher ratings of enjoyment in the VR condition [34]. VR is moving from a niche to a more affordable product, suggesting, for the first time, potential for the use of VR as a population health intervention. Ownership is increasing: Statista reported worldwide sales of VR headsets totaling 13.5 million units in 2017, and the sales are predicted to increase drastically by 2020 because of decreases in price [35].

### Objectives

VR exergames are a unique platform for PA intervention, and not much has been reported about the experiences of players themselves. This study aimed to investigate the factors that were reported to help or hinder engagement with VR exergames as reported by the players themselves in Web-based VR marketplaces or platforms. This review was performed to learn more about their actual experiences and synthesize results to be mindful when building a new VR exergame for PA. In line with intervention development frameworks, gathering the views of target users is crucial [32]. Some feedback can be obtained only from people who are using the technology in question, even though they may not be representative of all future users. Markets are changing rapidly and are future oriented, and the insights that we gather from current users may benefit the development of future VR PA interventions. The systems need to be well designed to be as effective as possible and preferably with an involvement with the target population [36].

New data collection could have been conducted with players of VR exergames, but we instead opted to take advantage of Web-based reviews that have been spontaneously submitted. Using material submitted Web-based has become increasingly common in research, and we drew on examples in app research [37]. This approach is quick and cost-effective. Web-based reviews may not directly answer researchers’ questions, and those submitting reviews may not be representative. However, Web-based reviews may have greater validity than responses given in a research context.

## Methods

Main gaming databases were searched in July 2018. Steam, Viveport, and Oculus were selected as the 3 main marketplaces for VR games and gaming experiences. Steam is the most popular hub in the world for purchasing and downloading desktop and VR games [38]. At present, Steam supports HTC Vive, Oculus Rift, and Windows Mixed Reality headsets [39,40]. Viveport and Oculus support their respective VR headsets. All 3 marketplaces encourage users to share their reviews.

### Virtual Reality Exergame Search

This study included games that were specifically marketed as being fitness related. Games that related to PA or sport but did not involve movement to play (eg, football management) were excluded. Each marketplace had slightly different game selection categories and or filter options, and these were applied to obtain a selection of VR exergames. The search strategies were as follows:

1. Steam: In the “Virtual Reality” category, “sort by Top Sellers” was selected and manually searched for fitness-related games.
2. We also visited the “Virtual Reality” category and selected “sort by What’s Being Experienced” (ie, hours played) and manually searched for any fitness-related games that were missed in the previous search.
3. Viveport: We searched for apps in the “Health & Fitness or Sports” genre, filtering by “Popularity”.
4. Oculus: We searched the fitness-related games in the categories “Top-selling experiences for Rift” and “Top Free experiences for Rift.”

### Types of User Reviews and Sampling

For each of the sampled VR games, 2 researchers (NF and AF) extracted the most recent 10 positive and 10 negative reviews. The reviews were extracted into an Excel sheet.

Steam and Oculus reviews require users to post either a positive or negative review. Viveport allows reviewers to post reviews using a starring system, ranging from 1 to 5. For all 3 marketplaces, we selected the first 10 reviews (chronologically most recent). This posed an issue of whether to include reviews with 3 stars (which would constitute as *neutral* and not necessarily a *positive* or *negative* review). We decided to include these because Steam and Oculus platforms, in fact, included reviews that would fall under a *neutral* category if such options existed. For example, in Steam and Oculus, some reviewers provided a breakdown and a list of *pros*, *neutrals*, and *cons* within one review, whereas others directly reported how many stars or categories they would assign if such options were available: “I could not give a positive review and there’s no neutral ones”, and “I’m a bit torn on reviewing this [before listing both pros and cons and arguing it was a neutral review].” It was merely the fact that the review could not be posted unless they have chosen either a positive or negative category, but in either of the categories, they have reported what would otherwise constitute a neutral review.

### Data Analysis

A thematic analysis was used as it provides a flexible approach to analyzing qualitative data [41]. We chose the thematic analysis over other diverse qualitative approaches because it helped us locate themes and patterns or *thematize meanings* within the body of data. Themes were organized in detail in a coherent manner [41]. The theoretical position of the thematic analysis in our study is concerned with a particular context and as such fell under a *contextualist* method or *critical realism*. It reports on the reality and experiences of the VR exergame users but at the same time unravels the way in which individuals make meaning of their experiences.

## Results

### Overview

The number of reviews extracted from each database is shown in Table 1. A total of 36 games were identified, but 7 of these had no associated reviews, leaving 29 games for inclusion. These produced a total of 498 reviews (260 positive, 238 negative, and 2 neutral reviews, ie, 3 stars for Viveport only). Table 2 gives details of the included games.

The VR exergames included in the analysis covered a wide variety of sport simulators (eg, Racket Fury) and super hero (eg, Echo Arena) or arcade-style games in VR (eg, Fruit Ninja). Of 498 reviews, 85 (17%) had both positive and negative comments within one review. This was apparent from the content of the reviews, and reviewers often separated feedback into defined paragraphs with *pros*, *neutrals*, and *cons* as subtitles in one review, as mentioned earlier. Approximately 70% of reviewers commented on the technological aspects of the games that included the settings and options in the games, the bugs, display functions such as the glitches and freezes, the overall flow of the game, the use of the controllers, and other potential issues (eg, the game requiring users to jump or spin while being potentially hazardous, eg, cables and space requirement).

Each review varied in length from 5 to 880 words. In terms of the context of this study, the word *workout* was mentioned 112 times in all reviews; *sweat* or *sweaty*, 61 times; *fitness*, 14 times; and *physical activity*, twice.

We identified 14 main themes for both positive and negative reviews, which we illustrate along with supporting quotes and summarize in Table 3.

**Table 1:** Selection of virtual reality exergames based on the marketplace.

Marketplace, game types		Number of games identified
**Steam**		
	Top sellers	12
	What’s being experienced (hours played)	6
**Viveport**		
	Health and fitness or sports genre (ordered by popularity)	10 (7 had no reviews)
**Oculus**		
	Top selling experiences for Rift	7
	Top free experiences for Rift	1

**Table 2:** Final virtual reality exergame selection and description of each virtual reality exergame.

VR^a^ exergame		Brief description of the VR exergame by marketplace (descriptions taken from or adopted from the marketplaces)
**Steam**		
	1—Beat Saber	The goal is to make players almost dance while cutting the cubes flying toward them and avoiding obstacles using 2 sabers. Each cut is strongly supported by great sound and visual effects to emphasize the rhythm
	2—Audioshield+C9	The players need to block the beats by punching the orbs flying toward them. Audioshield puts players at the point of impact for every hit in the music. Works with any song
	3—Sprint Vector	Run, jump, climb, drift, and fly at extreme velocity as you race up to 8 players and battle obstacles in this frenetic VR adrenaline platformer
	4—Hover Skate VR	Physics-based hoverboard simulator for VR with over 250 skateboard tricks to learn and master
	5—VR Super Sports	8 sports games combined into one
	6—Climbey	A VR-only climbing game! The goal is to climb to the finish as fast as you can, avoiding obstacles and trying not to fall along the way
	7—Soundboxing	A VR music video kickboxing game where you create the beats! You can either punch to the music and compete to beat the best, or record your own challenge for others to beat—all set to your favorite music in a cool outdoor environment
	8—BoxVR^b^	Rhythm-based boxing-inspired workouts. The only VR workout app with regularly updated workouts by professional fitness instructors
	9—The Thrill of the Fight—VR Boxing	A room-scale VR boxing game. Face off in the virtual ring where you’ll jab, dodge, and sweat your way to the top of the boxing world. Grab your gloves, step through the ropes, and become a champion
	10—Racket: Nx	Racquetball meets breakout inside a giant pinball machine! Racket: Nx is a new kind of game that challenges the limits of what you can do with a racket and ball in VR
	11—Racket Fury: Table Tennis VR^b^	Face 16 opponents in 4 distinctive cups and step into the most intense table tennis experience! Take on challenging rivals in the single-player campaign to climb the ladder of the Crown Galaxy! Get involved in heated exchanges with players from all over the world who, just like you, are hungry to win
	12—Holopoint	An archery game. Fight your way through waves of responsive targets, samurai, and highly dangerous ninjas—all while drawing, nocking, and shooting your arrows as quickly as possible
	13—Fruit Ninja VR	Step inside the Fruit Ninja universe and experience a slice of VR like never before
	14—Knockout League—Arcade VR Boxing	A single-player arcade-style boxing game built from the ground up for VR. Dodge flaming uppercuts, block sweeping tentacle attacks, and pummel your opponents using 1:1 tracking of your head and hands
	15—Eleven: Table Tennis	The ultimate table tennis simulator. Play opponents in Web-based multiplayer or practice against the advanced AI^c^. With physics designed to be as real as ever achieved in a Table Tennis simulator, you will forget you are in VR
	16—To the top	Play opponents in Web-based multiplayer or practice against the advanced AI. With physics designed to be as real as ever achieved in a Table Tennis simulator, you will forget that you are in VR
	17—Fancy Skiing VR	The game’s structure is based on real skiing that players use the handles just as ski poles to get power and lean left or right to control the direction, which brings players a strong immersion
	18—Sparc	A unique physical sport only possible in VR in which players compete in fast-paced, full-body VR gameplay and connect in a Web-based community
**Viveport**		
	19—Arcade Saga	Allows you to take the role of a newly sentient being and battle for survival against AI Overlords determined to destroy you and your freethinking. Through 3 different futuristic sports—Fracture, Smash, and Bowshot—you’ll harness your reactions and strategic thinking to shoot and dodge viruses, break firewall bricks, and spin data-balls past the Overlords and their minions. You will need to use your reflexes, wits, and changing strategies to survive
	20—VR Cricket	A cricket game for VR with 2 bowling options, that is, fast and spin. Game also features the real pitch and 2× dimensions of the real pitch. Game features a stadium, sound effects, and animations
	21—Knockout League^b^	A single-player arcade style boxing game built from the ground up for VR. Its intuitive gameplay involves moving and dodging with your body and 1:1 punch movement that lets you attack how you want without relying on buttons for main gameplay. Train up with various boxing drills to take on a crazy cast of characters with different fighting styles and gameplay to become the champion of the Knockout League
**Oculus**		
	22—Eleven: Table Tennis VR	The ultimate table tennis simulator. Play opponents in Web-based multiplayer or practice against the advanced AI. With physics designed to be as real as ever achieved in a Table Tennis simulator, you will forget you are in VR
	23—The Climb	Scale huge heights and feel the exhilaration of extreme free solo climbing. Explore and enjoy the view or compete for the fastest times on leaderboards with touch or gamepad controls
	24—BoxVR^b^	See game 8
	25—Knockout League^b^	See game 21
	26—Fruit Ninja VR	Fruit slicing game in VR. Step inside the Fruit Ninja universe and experience a slice of VR like never before
	27—Racket Fury: Table Tennis VR^b^	See game 11
	28—Streetball VR	Arcade-style basketball when you step into the street one on one with the top finest in basketball action and play in different streets with beautiful backgrounds in VR
	29—Echo Arena	Echo VR is your gateway to Echo Arena and Echo Combat. You play as a futuristic battle-ready robot armed with an array of weapons and abilities as your team of 3 competes against the opposition in a zero-gravity clash of robotic glory as you glide, boost, and punch your way to scoring goals in a breath-taking virtual arena

^a^VR: virtual reality.

^b^Where game repeats but in a different marketplace.

^c^AI: artificial intelligence.

**Table 3:** Main themes for both positive and negative virtual reality exergame reviews.

Themes	Description
Real-world feeling	Body movement akin to real-world sport or activities made games more appealing
Intuitive controls	Intuitive controls made games more playable and less frustrating, which was especially true for the less experienced players
Immersion	Immersion was important for sense of presence and overall enjoyment of the game
Music	Music greatly enhanced the overall experience of the games
Matching skill level	Gradual step-by-step acquisition of skill and skill-matched intensity levels highly impacted on playability
Social aspects	Sociable aspects of the games were favored, but sometimes multiplayer games left players feeling lonely or stuck
Exertion and matching real-world exercise levels	VR exergaming provides a high level of exertion, equivalent to real-world exercise
Appearance	The feeling of how you look when you play impacted on player’s enjoyment of the game
Graphics^a^	Good graphics and display allowed players to play for longer periods of time
Bugs^a^	Bugs frustrated the players, but when they were fixed quickly, people were more likely to play on
Add-ons enhance gaming experience	Simple add-ons and tweaks had the potential to enhance the overall game experience
Motion sickness^a^	Motion sickness made it difficult for players to engage
Developers	Responsive and present developers gained more players and praise
Price	Price influenced the overall perception of the VR exergames

^a^Main themes of the negative reviews, although also present in positive reviews

### Body Movement Akin to Real-World Sport or Activities Made Games More Appealing

People purchased and engaged with VR exergames for different reasons. Some people used VR exergames merely as entertainment, whereas others used them to build a skill, practice sport, and replace real-world sport or exercise. Sometimes VR exergaming did not match the feeling of performing sport or PA in the real world, and this had to do with controllers, mechanics (ie, the overall gameplay, sometimes interchangeably used as physics), physics (ie, simulation or game effects appearing to be realistic), or display quality and graphics of the game. These themes are interconnected. Reviewers often disagreed about the same game:

I’m a martial artist, I think this game is the closest to what we could get fighting in VR at the moment. If you would like a good work out, try playing ten rounds daily. It cannot replace actual boxing training but it will raise your confidence and fitness.

With all the good reviews, this must just not work with my system. The hit detection was horrible, couldn't block a thing, and it was constantly sticking me on top of my opponent.

Matching the physical skill levels meant that some exergames offered different modes of playing. Transferring the same physics to the VR exergames as those experienced in real-life sports or exercises was quite important for most players:

The most important aspect of table tennis is the physics and [name of the game] certainly ticks that box. It provides one of the best VR table tennis physics I have experienced to date. I have played over 240 hours of VR table tennis and play regularly in real life so I have a good idea of how the game of table tennis plays. [name of the game] translates so well that I can play all the shots that I play in real life with the correct amount of spin, power and accuracy. It is so accurate in fact that I can use it to improve my real game.

It’s designed for people who have never skated (it seems) whereby people that have will find it nonintuitive. Not sure if there’s a middle ground but I hope you find it (or add in an option for “skater mode” [“skater mode” would be a difficulty and skill level for more experienced skaters]).

### Intuitive Controls Made Games More Playable and Less Frustrating, Which Was Especially True for the Less-Experienced Players

Intuitive controllers affected playability and overall satisfaction with the game:

Braking requires hitting both side buttons, which is weird and uncomfortable (I’ve always hated those side buttons). All the buttons are annoying to remember and I can’t count the number of accidental screenshots I’ve taken.

The controls are incredibly simple, too: throw punches by pushing your hands out in front of your while holding the controllers; block by raising your hands in front of your face. There’s no need for button presses, as [name of game] is entirely based on movement.

### Immersion Was Important for the Sense of Presence and Overall Enjoyment of the Game

Intuitive controllers had an effect on immersion, but immersion also depended on other factors such as the mechanics of the game. The overall *flow* of the exergame was often related to immersion but was interrupted by poorly designed controllers or bugs:

Backstory is nonexistent. If you’re going for this style of game at least have a setup. Does wonders for immersion and gameplay. Trust me... [the name of an example game] made the game 10× better for me.

Especially when you complete a level and you’re at the peak. I usually sit there for 10 minutes taking in the view. The world is full of life, you can see boats and planes off in the distance. I also use wrist weights while playing, it enhances the immersion and its exercise.

The ball doesn’t follow your hand and instead leaves several seconds after you throw. This and a few other issues related to lag. Unfortunately it kills the immersion completely.

### Immersion Was Important for Distraction From Thinking About Physical Activity

One of the key features of VR exergaming is immersion, and immersion was reported as helping the players perform PA:

It’s a great workout and time flies while you tell to yourself: just one more song!

I’m a nerd. I hate sports and physical activity with a passion[...] I played until my body just couldn’t go on any more. As I’m writing this, I’m a sweaty mess and my whole body is hurting. The cross platform play is awesome, players are super nice to each other and the gameplay mechanics just work and the inputs are responsive [...] Now I need to go rest...

I also use wrist weights while playing, it enhances the immersion and its exercise.

### Music Greatly Enhanced the Overall Experience of the Games

Reviewers explicitly mentioned music. Most reviews referred to music directly in terms of enhancing the experience of playing satisfaction, immersion, replayability, and presence:

Music is excellent for the game’s style. I love this soundtrack.

Not a big fan of the music so would appreciate some audio controls.

Even though the songs are far from the kind of music that I listen to, I cannot help but walk around having the melodies in my head constantly. Some songs I play because I like the music, others I play because the level is just really fun to play. And some because both of those reasons.

Sometimes music hampered the enjoyment of the game entirely:

[name of the game] is a music game where the gameplay doesn’t match with the music. Not worth the price, especially now that [name of a competitor game] is out.

### Gradual Step-by-Step Acquisition of Skill and Skill-Matched Intensity Levels Highly Impacted on Playability

Some gamers spent long periods of time learning the mechanics of the game and commented on the technical support present in the game when they were stuck:

Great progression of levels keep the game interesting and require building new skills.

Good workout but overall, it jumps to ridiculously hard after 5 levels. I have been working on it for the last 2 days and I can’t get to round 10 because you get hit twice and it starts you over. The levels don’t even seem much different from 5-9.

### Sociable Aspects of the Games Were Favored, But Sometimes Multiplayer Games Left Players Feeling Lonely or Stuck

Fun was also dependent on whether players could play with other people such as friends and family but when players had no one to play against, which defeated the gameplay:

You & friends can even just hangout and chat & play in the skate park and not do races at all. Love the freedom.

This could be the best VR game so far. I love the game play. But the matchmaking wait is so bad - you have to wait and watch for 2.5 minutes between every game.

I live in Tokyo...my son is working in New Jersey...we met up in [name of the game] for over 2 hours and had a blast. As if he was next to me...we could actually grab hold each other’s hands to climb up the ledge.

### Virtual Reality Exergaming Provides a High Level of Exertion, Equivalent to Real-World Exercise

As already illustrated in the previous examples, the players commented on the level of exertion and workout quality. Additionally, some reviewers felt worried about their headsets getting sweaty and sought advice about maintenance and cleaning:

This game made me realise I am really out of shape.

This game is an amazing workout. It’s great for working the legs and your core (some arms too, but not nearly as intense). Right now I used 2 games [within the game] for exercise.

I was sweating on my head mounted display and just had to quit. Not only that, but I was tripping over my wired headset. If you have a wireless headset, then this game is perfect. If you don’t, you’ll have to setup your wires or just be very cognizant of this problem.

### The Feeling of How You Look When You Play Impacted on Players' Enjoyment of the Game

Some players wanted to look as cool to someone else when playing the game as they do inside the game itself. This was especially true for those games that offered superhero qualities or exceptional skills:

You shoot down holograms and dodge the projectiles. It is one of the few games where you actually look cool in the real world while you play it as you contort your body to dodge and shoot.

My wife thought I looked dumb shooting invisible arrows straight up at the ceiling while kneeling and swearing. I didn’t. I felt like I looked awesome. You will be ducking, kneeling, spinning, nocking, shooting, nocking, shooting until you fall over.

### Good Graphics and Display Allowed Players to Play for Longer Periods of Time

Clear, solid graphics made players like the exergames more, but sometimes too much detail evoked discomfort:

Graphics and models are well designed. Good amount of detail, but not so much that things are ever a pixelated aliased mess at a distance.

The graphics are terrible for a game this simple. Everything is shimmering and my eyes started to hurt soon after I started playing and made me uncomfortable.

### Bugs Frustrated the Players, But When They Were Fixed Quickly, People Were More Likely to Play On

Bug complaints were the most prevalent mechanics complaint across all reviews and for all games. VR technologies, hardware, and software issues were all mentioned. Some games were experienced as smooth, whereas others as unplayable:

Amount of bugs is recent versions is insane. Glitches, freezes control issues. Happen exclusively in this game. I wish there was a way to revert everything to the previous version.

It’s kind of frustrating to play through the song over and over again and not get a chance to really practice the hard part before you have to restart the song again.

Within minutes of use, I ran into several bugs and had to restart the game several times before I could even try it. So my first 10-15 minutes are just troubleshooting.. fun..

All the bugs are possible to work around and this kind of game lives on community engagement anyway.

Annoying bugs. Love the games but I hate the “Your head is intersecting with an object. Please move it” screen. It just randomly pops out when I fall making it impossible to grab on to a grip to the one beneath it [...] also, It keeps making me jump out of the middle of nowhere. So annoying.

### Simple Add-Ons and Tweaks Had the Potential to Enhance the Overall Game Experience

Sometimes reviews on mechanics had to do with simple add-ons or tweaks that helped players track fitness-related behaviors and PA:

Hours logged do not necessarily reflect all time spent in the game.

Need only to add more boxers and rings. Please add a calorie counter.

### Motion Sickness Made It Difficult for Players to Engage

Motion sickness was reported as problematic when playing certain VR exergames:

I am sometimes susceptible to VR sim sickness from artificial locomotion, depending on the game. [the name of the game] didn’t give me any problems.

I’ve got hundreds of hours in VR at this point, and no game has ever really made me sick the way [name of the game] has. I put on the comfort options in the game, but I still can't run more than one (maybe two) challenges before I have to leave the game.

### Responsive and Present Developers Gained More Players and Praise

Many reviews have tips and feedback for developers from praise to critique. Sometimes the developers were addressed directly and feedback was given to them about their response, behavior, or their presence Web-based, but sometimes the feedback was given to the developers more generally in terms of how they could improve the game mechanics or game controls:

Hats of[f] to the developers for the immersive experience and environments! Job well done! The touch controllers and style of locomotion are a great pairing.

I have been unable to launch the game for a few months now. I am extremely disappointed with the services in effort to help my problem and think that the services to help problems such as mine need to be looked at further by the developers of the game.

### Price Influenced the Overall Perception of the Virtual Reality Exergames

Reviewers commended or complained about the price. Some reviews described a greater degree of tolerance for glitches and bugs if the game price was low. Sometimes a low price meant that reviewers gave it a more balanced rating. The opposite was also true: reviewers expected more when the price was high:

Not nearly enough content in the game to justify the current price.

I’ll probably buy it again after they work out some of the bugs, or if the price comes down significantly.

For this small price, I give it a go!

## Discussion

### Principal Findings

This study sought to learn from users’ reviews of VR exergames from the 3 most popular marketplaces: Steam, Viveport, and Oculus. A thematic analysis revealed that VR can provide an immersive experience where the user feels fully present in the virtual environment and can provide a distraction and alternative to conventional PA while still providing exercise at an intensity equivalent to real-world PA. However, poor game design can frustrate players and lead to nonuse.

Players preferred games that are realistic (eg, closely simulated real-world sport); games that are intuitive (in terms of the body movement and physics of the game, game mechanics, and controls); games that provide gradual step-by-step increases in skill acquisition; games that either provide support (eg, tutorial) or social support; and games that provide access to accessories or player options. Concordant with a 2011 review of the literature on exergames, which emphasized how exergames can help physical education courses and improve a student’s physical and social outcomes, our study also suggested exergames to be enjoyable technology that increase energy expenditure during play, motivate players to become more physically active, and promote social interaction [42]. We were not able to measure or discern a player’s cognitive performance before and after playing VR exergames, but the aforementioned review also suggested exergaming (but not exclusively VR exergaming) to have the potential for positive cognitive effects such as problem solving, estimation, pattern recognition, memory, and improved academic performance [42]. Music was a popular choice to increase fun, immersion, and enjoyment of VR exergames. Players did not discuss weight loss but discussed fitness levels achieved as part of playing the VR exergames. Reviewers consistently reported that VR exergaming was providing a high level of exertion equivalent to real-world exercise and that the immersion and enjoyment was a welcome distraction from the exertion. Discussions centered on playability and enjoyment of the VR exergames and the experience of playing them.

A number of players did not feel that they were getting value for money. The overall experience with the VR exergame was tied to the price of the game. Some games were judged as worth the price, and some games were acceptable and more enjoyable because they were offered at a discounted rate. More was expected from games with higher price points.

There were 3 themes in negative reviews: the first was around bugs that rendered games frustrating and sometimes unplayable. The quality of graphics had a particularly strong impact on perceived enjoyment. Reviewers disliked when games had overly complex controls and display functions that evoke motion sickness, which is a potential side effect in VR displays [43,44].

These negative effects clash with VR’s potential to improve mood, visual-spatial skills, coordination, motivation, and energy expenditure [34,42], and as such, careful consideration for the design features of the future exergames is paramount. What is particularly important is that the caloric expenditure, the end goal of the engagement with VR exergames, may be hampered by technological glitches and factors that can most likely only be modified by the designers of the games. Therefore, the first important factor is the design and consideration of the potential negative effects when designing a new VR exergame and taking user’s feedback on board. In doing this, the designers are more likely to create a game that will flow and then also promote the state of *flow*, a state of absorption in the activity [45], which is a key factor in the attractiveness of exergames. Other important factors include the balance of skill and the perceived challenge, clear goals and rules, fair feedback or guidance, a sense of personal control over the activity or game, loss of self-doubt or feelings of self-concern (loss of consciousness), transformation of time (losing sense of time and feeling absorbed), and autotelic experience (an activity undertaken for its own sake) [46]. The balance between making the game appealing to a wide variety of people when taking into account individual differences (eg, age and disability status) may mean accepting that a great variety of choices are required, even within the same exergame to keep the game challenging, fun, and novel [46]. Recently, attempts have been made to maximize individualization using interactive deep reinforcement learning, which allows the game to adapt to user preferences [47].

### Limitations and Future Research

This study has a sufficiently large sample size [48,49]. However, the user-submitted reviews may be biased because they could be from people who felt more strongly or passionately about the VR exergames or who had something very particular to say. There were no demographic data about reviewers or their experience of playing VR games, and as such, we cannot draw any conclusions about the characteristics, motivations, and overall VR experiences of reviewers.

Nor could we examine whether comments varied systematically by any individual differences between players. A study on affective and attentional states when running in a VR environment concluded that the effects of performing PA in a VR environment depend on individual difference factors (especially individual differences in presence and immersive tendencies) [50].

This study was an inductive thematic analysis with no a priori hypothesis or theory. It is difficult to draw conclusions with regard to whether these findings are transferable to all cultures or ages and if these findings were an optimal reflection of the VR exergaming community. However, this relatively novel approach to thematic analysis allowed us to capture the views of a large number of users, and it also complements traditional qualitative interviewing approaches.

### Conclusions

This thematic analysis indicates that VR exergames have potential as a public health intervention and gives specific advice on what a game should do (including the importance of price and building a relationship between developers and the gaming community—issues often overlooked in a research or public health context). More research is necessary to determine which factors matter most across a wide range of exergame genres in the VR gaming space. However, we believe that users and developers will benefit from our findings. However, further exploration of factors influencing long term engagement with VR exergames is warranted.

## Abbreviations

PA: physical activity
VR: virtual reality
